# Antibodies against atypical pathogens and respiratory viruses detected by Pneumoslide IgM test in adults with community‐acquired pneumonia in Guangzhou City

**DOI:** 10.1002/jcla.23419

**Published:** 2020-06-14

**Authors:** Sheng Qin, Weizheng Zhang, Fu Chen, Fudong Luo, Qiang Zhou, Peifeng Ke, Cha Chen

**Affiliations:** ^1^ Department of Laboratory Medicine The Second Affiliated Hospital of Guangzhou University of Chinese Medicine Guangzhou China

**Keywords:** atypical pathogens, community‐acquired pneumonia, Guangzhou City, pneumoslide IgM test, respiratory viruses

## Abstract

**Background:**

To detect the serum antibodies against respiratory viruses and atypical pathogens in adults with community‐acquired pneumonia (CAP) in Guangzhou City (Guangdong province, China).

**Methods:**

A retrospective study was carried out with samples from 685 adults who were admitted with CAP and 108 non‐CAP control patients. Atypical pathogens and respiratory viruses in serum were detected using the Pneumoslide IgM test from Vircell, Spain. All patients were divided into 6 groups according to age: 18‐24, 25‐44, 45‐59, 60‐74, 75‐89, and >90.

**Results:**

The total positive rate of CAP was 35.4%, which was highest in the 18‐24 age group (*P* < .05). The highest positive rate, 17.11%, was observed for *Mycoplasma pneumoniae* (MP). The mean age of MP‐infected patients was higher than that of the controls (*P* < .05). The positive rates for *influenza B* (INFB), *Legionella pneumophila* (LP1), *Coxiella burnetii* (COX), influenza A (INFA), parainfluenza virus (PIV), respiratory syncytial virus (RSV), *Chlamydophila pneumoniae* (CP), and adenovirus (ADV) were 5.56%, 3.07%, 2.63%, 2.34%, 1.90%, 1.61, 0.88%, and 0.29%, respectively. There were 4.37% of patients with CAP having multiple infections. The main symptoms observed in the 685 CAP patients were cough and sputum production, in 78.4% and 67.4%. Fever was followed by 54% of CAP patients. Dyspnea (39.1%), anorexia (36.8%), increased thirst (26.7%), chills (18.7), headache (14.6%), and nausea (13.1%) were also frequently observed in the CAP patients.

**Conclusions:**

MP infection was the most common in adult CAP patients in Guangzhou City with the highest positive rate in the 18‐24 age groups.

## 1 INTRODUCTION

Community‐acquired pneumonia (CAP) is a common infectious disease worldwide, with a mortality rate of 2%‐14%.^1^, ^2^, ^3^, ^4^, ^5^ Recently, it has been found that the importance of atypical bacterial pathogens and viruses in CAP has been underestimated.^6^, ^7^ Previous study has shown that some of CAP were caused by atypical bacterial pathogens and viruses.^8^ Jain et al studied 3634 American adults with CAP and found that the most common pathogens were human rhinovirus (in 9% of patients), influenza virus (in 6%), and *Streptococcus pneumoniae* (in 5%). Chen et al studied 1204 children with pneumonia and found that MP was the most dominant pathogen, followed by *influenza B* (INFB), parainfluenza virus (PIVs), and respiratory syncytial virus (RSV).^9^

Virus, as a cause of CAP, is more common in children than in adults.^10^, ^11^, ^12^ However, the study of its importance in adults is relatively insufficient.^13^ The assessments of the prevalence of viral infection in adults with CAP based on a large study population in China have only been performed in Beijing, Shanghai, and Jinan.^14^, ^15^, ^16^ With the understanding of atypical pathogens,^6^, ^7^ evaluations of their role in lower respiratory tract infections have been gradually increasing. However, reports of viruses and atypical pathogens in adults with CAP remain scarce.^17^, ^18^, ^19^

Currently, the greatest challenge in the diagnosis and treatment of CAP in China is the lack of etiological diagnosis in everyday clinical practice.^20^, ^21^ The available methods for etiological diagnosis of infection are serology, culture, and PCR.^22^, ^23^, ^24^ Pneumoslide IgM, an indirect immunofluorescent assay, allows the identification of IgM antibodies in human serum/plasma against the main etiological agents in respiratory infectious diseases (ie, Legionella pneumophila serogroup 1, *Mycoplasma pneumonia* (MP), *Coxiella burnetii*, *Chlamydophila pneumoniae*, adenovirus, respiratory syncytial virus, influenza A, influenza B, and parainfluenza serotypes 1, 2, and 3). This technique, which has been considered as a useful diagnostic tool for the detection of atypical pneumonia, has been approved by the China Food and Drug Administration (SFDA). It is currently utilized in general hospitals and community hospitals throughout China. To further understand the spread of the atypical pathogens and respiratory virus and the clinical characteristics in adults with CAP in Guangzhou City, a retrospective study of available laboratory and hospital admission data from adults with CAP was performed in the Guangdong Provincial Hospital of Chinese Medicine in Guangzhou.

## 2 PATIENTS AND METHODS

### 2.1 Patients

This study was reviewed and approved by the ethics committee of Guangdong Provincial Hospital of Chinese Medicine. Because this was a retrospective study of medical record data with no patient contact and no collection of personal data, the study was exempt from obtaining informed consent. The adult patients with CAP who were hospitalized in the Guangdong Provincial Hospital of Chinese Medicine between September 2014 and October 2015 were included in this study. Inclusion criteria were as follows: (a) The patient's age is older than 18 years old; (b) patients were identified as showing suspicion of acute respiratory tract infection with a new or progressive infiltrate on a chest radiograph; (c) fever (temperature ≥38°C), or hypothermia (temperature <35°C); and (d) patients with confusion/disorientation and the symptom of an acute respiratory illness, such as a new cough with or without sputum production, abnormal percussion and presence of fine crackles on auscultation, dyspnoea, tachypnoea, respiratory failure, and leucocytosis or leukopenia.

The unmatched controls (at a rate of 2‐3 persons per week) were randomly selected between September 2014 and October 2015 for the comparative purposes to obtain baseline rates. A total of 108 non‐CAP control adult patients were admitted to the same hospital for reasons other than respiratory symptoms or acute infection (eg, individuals with muscle sprains).

### 2.2 Sample collection

A total of 3 mL of venous blood were drawn from each patient within 24 hours after admission. The specimens were sent to the clinical laboratory immediately and were centrifuged at 11268*g* for 20 minutes at 4°C. The serum was obtained and stored at −20°C until assayed with the Pneumoslide IgM test.

### 2.3 Pneumoslide IgM test (Vircell, Granada, Spain)

A 1:2 dilution of serum samples were prepared with phosphate‐buffered saline (PBS) and treated with anti‐human IgG sorbent. Then, they were added to every well containing the Pneumoslide IgM slide, respectively. After incubating for 90 minutes at 37°C, the slide was washed twice with PBS and dried. The fluorescent secondary antibody was added and incubated at 37°C for 30 minutes. Then, the slide was washed twice with PBS and observed under the microscope.

Apple green fluorescence was observed in nuclear, cytoplasmatic, and/or peripheral in 1%‐15% of the cells for positive samples with adenovirus, influenza, VSR, or parainfluenza (peripheral pattern was most frequent with weak samples; in parainfluenza and VSR dyed syncytia together with the previous pattern can be observed). Apple green fluorescence in all the bacteria in the case of Legionella, Chlamydophila, or Coxiella can be observed. Apple green fluorescence in the periphery of the cell for positive samples to Mycoplasma can be observed. A negative sample showed no fluorescence for Legionella, Chlamydophila, and Coxiella and red cellular pattern for Mycoplasma, adenovirus, influenza A and B, VSR, and parainfluenza. The apple green fluorescent signal was detected using a fluorescence microscope. Antigens corresponding to each of the following pathogens were present in each slide: LP1, MP, COX, CP, ADV, RSV, INFA, INFB, and PIV 1, 2, and 3.

### 2.4 Statistical analysis

Statistical analysis was performed using the SPSS 17.0 software program (SPSS). Quantitative data were presented as the means ± standard deviation (SD). The categorical variables were expressed as frequencies and percentages and were compared using chi‐square or Fisher's exact test. Differences for the pathogen detection rates in the various groups were examined using the chi‐square test. The positive rates in different age groups were compared using the chi‐square test. Comparisons of the mean age between adults with the three most prominent pathogens and controls were performed by analysis of variance (ANOVA). *P* < .05 was considered to be statistically significant.

## 3 RESULTS

### 3.1 Demographics

From September 2014 to October 2015, 685 hospitalized CAP adult patients and 108 non‐CAP control patients were included in the study. The demographic characteristics of the CAP patients were shown in Table 1. There was no significant difference in gender and comorbidities (liver disease, cerebrovascular disease, hypertension, and diabetes mellitus) between the two groups. The age of patients with CAP was higher than that of control patients (68.77 ± 18.56 vs 60.9 ± 20.53) (*P* < .01). The percentage of kidney disease in CAP patients was significantly higher than that in control patients (18.8% vs 10.2%) (*P* < .05).

**Table 1:** Demographics of CAP patients and non‐CAP controls

Characteristics	CAP patients (n = 685)	Controls (n = 108)	*P* value*
Age (means ± SD)	68.77 ± 18.56	60.9 ± 20.53	<.01**
Range (years old)	16‐102	16‐96	
Gender [n (%)]			
Male	382 (55.7%)	57 (52.3%)	.49
Female	303 (44.3%)	52 (47.7%)	.27
Comorbidity [n (%)]			
Liver disease	118 (17.2%)	12 (11.1%)	.103
Kidney disease	129 (18.8%)	11 (10.2%)	<.05*
Cerebrovascular disease	90 (13.1%)	15 (13.9)	.859
Hypertension	311 (45.4%)	53 (49.1%)	.531
Diabetes mellitus	137 (20%)	24 (22.2%)	.626

Note

Comparisons were made using ANOVA for quantitative data and chi‐square or Fisher's exact test for categorical variables.

* *P* < .05 and

** *P* < .01 vs control.

### 3.2 Breakdown of the detected pathogens

The results were shown in Table 2. Among the 685 patients, the total positive rate was 35.4%. A positive rate of 17.11% was observed for MP, which was the highest among the nine examined pathogens. The next most commonly detected pathogen was INFB (5.56%). The detection rates of LP1, COX, INFA, PIVs, RSV, CP, and ADV were 3.07%, 2.63%, 2.34%, 1.90%, 1.61%, 0.88%, and 0.29%, respectively. The positive rate of LP1 in CAP patients was higher than that of controls (3.07% vs 0%). The positive rates of other pathogens were similar between the two groups (*P* > .05).

**Table 2:** Detection of pathogens in CAP patients and controls

Pathogens	CAP patients (n = 685)	Controls (n = 108)	*P* value^*^
*Legionella pneumophila* serogroup 1 (LP1)	21 (3.07%)	0 (0.0%)	.06
*Mycoplasma pneumoniae* (MP)	117 (17.11%)	21 (19.44%)	.33
*Coxiella burnetii* (COX)	18 (2.63%)	1 (0.93%)	.48
*Chlamydophila pneumoniae* (CP)	6 (0.88%)	0 (0.0%)	.60
Adenovirus (ADV)	2 (0.29%)	0 (0.0%)	1.00
Respiratory syncytial virus (RSV)	11 (1.61%)	1 (0.92%)	1.00
Influenza A (INFA)	16 (2.34%)	0 (0.0%)	.14
Influenza B (INFB)	39 (5.70%)	6 (5.56%)	.67
Parainfluenza 1, 2, and 3 (PIVs)	13 (1.90%)	2 (1.85%)	1.00

Note

Chi‐square or Fisher's exact test was used to compare the detection of each pathogen between CAP patients and controls. Data were expressed as n (%).

* *P* < .05 vs control.

### 3.3 Mixed infection modes of the pathogens

There were 30 patients in which two or more pathogens were detected, representing 4.37% of the samples, and the modes of mixed infection were complex (Table 3). Among the specimens shown infection with two pathogens, the MP + PIV mixed infection was the most common, with 5 cases. The next mixed infections were MP + INFA, MP + INFB, and MP + LP1, with 3 cases in each. In addition, 5 cases of co‐infections with three pathogens were also been observed in Table 3.

**Table 3:** Mixed infection modes of the pathogens

Mixed infection modes	Cases (n)
LP1 + MP	3
LP1 + INFA	2
LP1 + RSV	1
MP + PIVs	5
MP + INFA	3
MP + INFB	3
MP + CP	2
INFB + PIVs	1
INFB + COX	1
INFB + CP	1
PIVs + RSV	1
PIVs + INFA	2
LP1 + MP + INFB	1
RSV + INFA + PIVs	1
MP + COX + INFB	1
MP + RSV + ADV	1
MP + INFB + RSV	1

Abbreviations: ADV, adenovirus; COX, *Coxiella burnetii*; CP, *Chlamydophila pneumoniae*; INFA, influenza A; INFB, influenza B; LP1, *Legionella pneumophila* serogroup 1; MP, *Mycoplasma pneumonia*; PIVs, parainfluenza 1, 2, and 3; RSV, respiratory syncytial virus.

### 3.4 The positive rates of the pathogens isolated from different age groups

Overall, the positive rates for the 9 pathogens were 86.7% in the (18‐24) age group, 37.7% in the (25‐44) age group, 33.00% in the (45‐59) age group, 38.9% in the (60‐74) age group, 30.2% in the (75‐89) age group, and 38.2% in the (>90) age group, respectively. The positive rate of viruses and atypical bacterial pathogens was highest in the (18‐24) age group (*P* < .05). The positive rate of the 3 major pathogens, MP, INFB, and LP1, did not differ among the age groups (*P* > .05). However, compared with the controls, the mean ages of patients with MP and patients with CAP were higher (*P* < .05 and *P* < .01, respectively) (Table 4).

**Table 4:** Relationship between percentage positivity and age group

Pathogens	Age (years old)							Patient age (years old)		
	18‐24 (n = 15)	25‐44 (n = 69)	45‐59 (n = 100)	60‐74 (n = 167)	75‐89 (n = 278)	>90 (n = 55)	*P* value*	CAP patients	Controls	*P* value^†^
MP	5 (33.3%)	15 (21.7)	14 (14.0%)	31 (18.6%)	41 (14.7)	11 (20%)	.28	66.40 ± 20.57	54.33 ± 27.33	<.05
INFB	3 (20.0%)	5 (7.2%)	5 (5.0%)	10 (6.0%)	15 (5.4)	1 (1.8%)	.4	64.71 ± 20.71	49.16 ± 20.18	.093
LP1	‐	4 (5.7%)	6 (6.0%)	3 (1.8%)	7 (2.5)	1 (1.8%)	.27	62.38 ± 20.28	‐	‐
PIVs	2 (13.3%)	2 (2.9%)	1 (1.0%)	4 (2.4%)	0 (0.0)	3 (5.5%)	‐	‐	‐	‐
INFA	‐	‐	‐	8 (4.8%)	5 (1.8)	3 (5.5%)	‐	‐	‐	‐
COX	‐	‐	4 (4.0%)	4 (2.4%)	8 (2.9)	2 (3.6%)	‐	‐	‐	‐
CP	‐	‐	2 (2.0%)	2 (1.2%)	2 (0.7)	‐	‐	‐	‐	‐
ADV	1 (6.7%)	‐	‐	‐	1(0.4)	‐	‐	‐	‐	‐
RSV	2 (13.3%)	‐	1 (1.0%)	3 (1.8%)	5 (1.8)	‐	‐	‐	‐	‐
Total	13 (86.7%)	26 (37.7%)	33 (33.0%)	65 (38.9%)	84 (30.2%)	21 (38.2%)	<.01	68.77 ± 18.56	60.9 ± 20.53	<.01

Note

Data in different age group were expressed as n (%). Data in CAP and control group were expressed as means ± SD.

* Comparison of percentage positivity in different age groups by the chi‐square test.

^†^ Comparison of the mean age of patients and controls with the three most prominent pathogens by ANOVA.

### 3.5 Clinical characteristics and blood test values of CAP patients

The patients’ clinical characteristics were summarized in Table 5. The results revealed that cough and expectoration were the most common symptoms of CAP and were significantly more frequent in the CAP group (78.4% and 67.4%, respectively) in comparison of the control group (*P* < .01). A total of 310 patients with CAP had a body temperature more than 38°C, which was significantly different from controls, and 54% (169/310) of them had 0‐3 days of fever. In general, the symptoms of dyspnea (39.1%), anorexia (36.8%), thirst (26.7%), chill (18.7), headache (14.6%), and dizziness (13.1%) were also notably more common in the CAP group than those in the control group ((*P* < .01, respectively).

**Table 5:** Major clinical symptoms and blood test values of the 685 CAP patients

Symptoms	CAP patients (n = 685)	Controls (n = 108)	OR (95% CI)	*P* value*
HT ≥ 38°C	310 (45.3%)	9 (8.3%)	9.18 (4.56, 18.46)	<.01
Days of fever				
0‐1 D	80	‐	‐	‐
2‐3 D	89	‐	‐	‐
4‐5 D	65	‐	‐	‐
6‐10 D	51	‐	‐	‐
11‐15 D	9	‐	‐	‐
>15 D	19	‐	‐	‐
Chills	128 (18.7**%**)	6 (5.6**%**)	3.94 (1.69, 9.18)	<.01
Nausea	90 (13.1**%**)	26 (24.1**%**)	0.48 (0.29, 0.79)	<.01
Headache	100 (14.6**%**)	8 (7.4**%**)	2.15 (1.01, 4.57)	<.05
Myalgia	54 (7.9**%**)	5 (4.6%)	1.78 (0.69, 4.55)	.22
Fatigue	184 (26.7**%**)	21 (19.4%)	1.53 (0.92, 2.55)	.09
Rhinorrhea	46 (6.7**%**)	4 (3.7**%**)	1.89 (0.66,5.35)	.29
Nasal discharge	51 (7.4**%**)	3 (2.7%)	2.84 (0.87, 9.27)	.09
Thirst	183 (26.7**%**)	13 (12.0%)	2.69 (1.47,4.92)	<.01
Sore throat	62 (9.1**%**)	5 (4.6%)	2.07 (0.81, 5.27)	.11
Dry cough	11 (1.6**%**)	1 (0.9%)	1.76 (0.22, 13.79)	1.00
Cough	537 (78.4**%**)	25 (23.1%)	12.19 (7.52, 19.74)	<.01
Expectoration	462 (67.4**%**)	21 (19.4%)	8.68 (5.25, 14.34)	<.01
Haemoptysis	10 (1.5**%**)	2 (1.9%)	0.79 (0.17, 3.66)	.67
Chest pain	40 (5.8**%**)	2 (1.9%)	3.31 (0.79, 13.93)	.10
Dyspnea	268 (39.1**%**)	13 (12.0%)	4.74 (2.60, 8.64)	<.01
Vomiting	6 (0.9**%**)	2 (1.9%)	0.47 (0.09, 2.47)	.30
Anorexia	252 (36.8**%**)	23 (21.3%)	2.17 (1.33, 3.53)	<.01
Celialgia	23 (3.4**%**)	10 (9.3%)	0.34 (0.15, 0.74)	<.01
Blood test	Value (n = 665)	Value (n = 97)		
WBCs (×10^9^/L)	9.38 ± 6.6	8.66 ± 5.20	‐	.299
NEU (%)	75.52 ± 30.22	71.82 ± 15.00	‐	.237
LYM (%)	19.31 ± 13.38	16.34 ± 12.74	‐	<.05
MONO (%)	10.31 ± 31.68	7.39 ± 4.96	‐	<.05
HGB (g/L)	112.08 ± 26.62	113.66 ± 26.35	‐	.585
PLT (×10^9^/L)	194.18 ± 98.53	211.72 ± 123.52	‐	.114
HCT (%)	34.04 ± 7.30	34.57 ± 7.13	‐	.501

Note

Data in symptoms were expressed as n (%). Data in blood test were expressed as means ± SD.

Abbreviations: CI, confidence interval; HCT, hematocrit; HGB, hemoglobin; HT, highest temperature; LYM, lymphocytes; MONO, monocytes; NEU, neutrophils; OR, odds ratio; PLT, platelets; WBCs, white blood cells.

* ANOVA was used to compare the differences for quantitative data, and chi‐square or Fisher's exact test was used to compare the differences for categorical variables.

The laboratory results, which were obtained from 665 patients with CAP and 97 controls, demonstrated that the higher percentages of lymphocytes (LYM) and monocytes (MONO) had prognostic significance in patients with CAP (*P* < .05). However, there was no statistically significant difference in white blood cell (WBC) counts, neutrophils (NEU%), hemoglobin (HGB) levels, platelet (PLT) counts, or hematocrit (HCT) between patients with CAP and controls based on the blood test (*P* > .05) (Table 5).

## 4 DISCUSSION

Atypical pathogens and viruses constitute an important challenge in the management of patients with lower respiratory tract infection (LRTI) admitted to pediatric intensive care unit (PICU). To optimize the clinical diagnostic, a detection test for respiratory pathogens must be sensitive, fast, low‐cost, and capable to detect a wide range of pathogens.

In this study, Pneumoslide IgM test was used to detect approximately 685 patients with CAP. Our results suggested that the positive rate of total atypical pathogen and respiratory virus was 35.4%, which was similar to that of other studies.^11^, ^25^ There were no significant differences observed in the positive rates of a variety of pathogens in the CAP group compared with the control group. Those results showed that what was detected might be background. A second technique to compare to would be useful here. All samples were collected from individual patients, that is, duplicate samples were not taken from any patient.

In addition, we found that the age of patients with CAP was higher than that of controls. We speculated that the difference in age between patients with CAP and individuals with non‐CAP may be attributed to the increasing comorbidities and decreasing immunity with age. It indicated that older patients with atypical pathogens and respiratory virus infections may be more susceptible to CAP than younger patients.

In studies from China and other Asian countries, MP was the most common atypical pathogen detected in patients with CAP.^15^, ^22^, ^26^, ^27^ We detected MP in all age groups, and the results showed that patients with MP were older than controls, and there was a greater frequency of MP in the (18‐24) age groups. Those finding suggested that MP was an important CAP‐related pathogen in the (18‐24) age groups in patients in Guangzhou.

Viruses were detected in approximately one‐third of patients, which was in agreement with the 13%–56% range reported in previous studies.^5^, ^8^, ^28^ In agreement with these studies, our study revealed that the detection rate of viruses was not high in adult patients with CAP, which may be because the total number of antibodies was increased in adult sera with increasing age. It made memory T‐cell populations respond to pathogens that the body had encountered in the past. Positive influenza B viruses were mostly observed upon detection of the virus, which was consistent with the epidemic trend of influenza viruses in China during 2014‐2015.^29^, ^30^ We also found that influenza B viruses had a greater positive rate in the (18‐24) age group (20%). The positivity rates of the atypical pathogens *Mycoplasma pneumoniae*, *Legionella pneumophila*, and *Chlamydia pneumoniae* were consistent with the results of worldwide atypical pathogen detection in a foreign study.^31^, ^32^

Paranhos‐Baccala et al reported that according to clinical research, co‐infections were more severe than single infections.^33^ The proportions of mixed infections reported in adults with CAP range from 5.1% to 10.5% in China.^15^, ^16^, ^26^ This is due to diverse pathogens, test methods, and study designs. In our study, 4.37% of the patients were infected with multiple pathogens, and there were a large number of patients infected with MP combined with another atypical pathogen or virus. Those results were similar to those in other studies from China.^15^ This result suggested that patients with MP infection may be susceptible to infection with other pathogens, resulting in mixed infections, such as MP with INFB or MP with PIVs. Whether infection by one agent facilitates infection by the same pathogen or other pathogens in cells was still uncertain. Some studies suggested that extensive damage to the epithelium of the respiratory tract in some viral ARTIs may promote superinfection by another virus.^34^ When treating a mixed infection, particular attention must be paid to the characteristics of the infection and the responsible pathogens.

Some studies have described the associations of chronic kidney disease (CKD) and acute kidney injury (AKI) with the incidence of CAP among older people.^35^, ^36^ In our study, we found that patients with pre‐existing kidney disease were at a higher risk of developing CAP. Whether this relationship is causal is less clear, and we believe that potential mechanisms include immune system dysfunction.

There were also some limitations in this study. There was unequal number of patients in both groups. The detection methods of atypical pathogens and respiratory viruses were signal. More than one detection methods should be performed in further study.

Based on the clinical characteristics observed in this study, clinicians should remain on high alert for patients who may suffer from CAP if they express the following symptoms: highest body temperature over 38°C within 3 days of onset, cough, expectoration, dyspnea, anorexia, thirst, chill, headache, dizziness, and changes in pulmonary consolidation signs. However, these differences are clinically insignificant unless combined with the identification of atypical pathogens or respiratory viruses.

## 5 CONCLUSIONS

In conclusion, our results suggested that MP was the most common causative pathogen in adult patients with CAP and had the highest positive rate in the (18‐24) age groups. Compared with the controls, those with pre‐existing kidney disease were at a higher risk of developing subsequent CAP. The patients with CAP characterized by cough, expectoration, and fever exhibited obvious systemic and respiratory symptoms. The MP infection in patients with 18‐24 years old should be paid more attention to in further study. Furthermore, the study on the etiology and clinical characteristics of adults with CAP in Guangzhou will not only help to determine the etiology of CAP in this area, but also assist in improving the levels of disease diagnosis, treatment, and management in different age groups.

## ETHICAL APPROVAL AND CONSENT TO PARTICIPATE

This study was reviewed and approved by the ethics committee of Guangdong Provincial Hospital of Chinese Medicine. Because this was a retrospective study of medical record data with no patient contact and no collection of personal data, the study was exempt from obtaining informed consent.

## CONSENT FOR PUBLICATION

Not applicable.

## Data Availability

Not applicable.
